# Analysis of the Dynamics of Infiltrating CD4^+^ T Cell Subsets in the Heart during Experimental *Trypanosoma cruzi* Infection

**DOI:** 10.1371/journal.pone.0065820

**Published:** 2013-06-11

**Authors:** Cristina Sanoja, Sofía Carbajosa, Manuel Fresno, Núria Gironès

**Affiliations:** 1 Instituto de Biología Experimental, Universidad Central de Venezuela (UCV), Caracas, Venezuela; 2 Centro de Biología Molecular Severo Ochoa (CBMSO), Consejo Superior de Investigaciones Científicas (CSIC)–Universidad Autónoma de Madrid (UAM), Cantoblanco, Madrid, Spain; 3 Instituto de Investigación Sanitaria Princesa (IP), Madrid, Spain; Albert Einstein College of Medicine, United States of America

## Abstract

Chagas disease, caused by the protozoan parasite *Trypanosoma cruzi*, affects several million people in Latin America. Myocarditis, observed during both the acute and chronic phases of the disease, is characterized by an inﬂammatory mononuclear cell infiltrate that includes CD4^+^ T cells. It is known that Th1 cytokines help to control infection. The role that T_reg_ and Th17 cells may play in disease outcome, however, has not been completely elucidated. We performed a comparative study of the dynamics of CD4^+^ T cell subsets after infection with the *T. cruzi* Y strain during both the acute and chronic phases of the disease using susceptible BALB/c and non-susceptible C57BL/6 mice infected with high or low parasite inocula. During the acute phase, infected C57BL/6 mice showed high levels of CD4^+^ T cell infiltration and expression of Th1 cytokines in the heart associated with the presence of T_reg_ cells. In contrast, infected BALB/c mice had a high heart parasite burden, low heart CD4^+^ T cell infiltration and low levels of Th1 and inflammatory cytokines, but with an increased presence of Th17 cells. Moreover, an increase in the expression of IL-6 in susceptible mice was associated with lethality upon infection with a high parasite load. Chronically infected BALB/c mice continued to present higher parasite burdens than C57BL/6 mice and also higher levels of IFN-γ, TNF, IL-10 and TGF-β. Thus, the regulation of the Th1 response by T_reg_ cells in the acute phase may play a protective role in non-susceptible mice irrespective of parasite numbers. On the other hand, Th17 cells may protect susceptible mice at low levels of infection, but could, in association with IL-6, be pathogenic at high parasite loads.

## Introduction

Chagas disease, caused by the protozoan parasite *Trypanosoma cruzi*, affects approximately 10–12 million people in Latin America and kills more than 15000 each year, thus representing a major cause of morbidity and mortality in this region [1]. Myocarditis is the most serious and frequent manifestation of chronic Chagas disease and appears in 30% of infected individuals several years after infection occurs. The pathogenesis is thought to be dependent on an immune-inflammatory reaction to a low-grade infection [2], [3]. *T. cruzi* has a complex life cycle involving several stages in both vertebrates and insect vectors. It infects and replicates in both macrophages and cardiomyocytes as well as many other cell types.

There is evidence that the CD4^+^ T helper (Th)-1 response mediated by Interferon (IFN)-γ is protective against infection *in vitro* [4], [5] and *in vivo* [6], [7]. On the other hand, regulatory T (T_reg_) cells may help to control T cell responses during infection. Natural (n)T_reg_ cells develop in the thymus and help to maintain self-tolerance [8]. T_reg_ cells can also be generated in the presence of interleukin (IL)-2 and transforming growth factor (TGF)-β or as induced (i)T_reg_ cells in response to infection by microorganisms. T_reg_ cells are characterized by the expression of both CD4 and CD25, the transcription factor forkhead box P3 (FoxP3) and some also produce IL-10 and/or TGF-β [9]. Nevertheless, T_reg_ cells constitutively express high amounts of the folate receptor (FR)4 [10] and may lose CD25 expression [11]. T helper (Th)17 cells characterized by IL-17 production, are pro-inflammatory cells associated with autoimmune diseases [12] Reciprocal developmental pathways have been described for the generation of both T_reg_ and Th17 cells, with Th17 requiring both TGF-β and IL-6 for differentiation [13].

The role of T_reg_ and Th17 cells in *T. cruzi* infection is not completely understood. Peripheral T_reg_ cell numbers were higher in patients during the indeterminate phase of Chagas disease in comparison with patients with overt cardiac pathology [14], [15], [16], suggesting that the regulatory response plays a protective role. Studies on T_reg_ depletion with anti-CD25 antibodies in acute and chronic mouse experimental models involving highly susceptible mouse-parasite strain combinations (C57BL/6-Tulahuén strain [17], [18] or BALB/c-Y strain [19]), have however, suggested a limited role for T_reg_ cells in the control of *T. cruzi* infection.

On the other hand, deficient regulatory T cell activity and a low frequency of IL-17-producing T cells have been correlated with cardiomyopathy in human Chagas disease patients [20]. IL-17 has been shown to play a protective role against parasite-induced myocarditis in BALB/c mice infected with the Y strain, by inhibiting Th1 differentiation during the acute phase of infection [21]. IL-17 has also been shown to confer systemic protection against infection by mediating neutrophil recruitment in C57BL/6 mice infected with the *T. cruzi* Tulahuén strain [22], [23]. Thus, different mechanisms seem to mediate protection depending on the mouse model, the *T. cruzi* strains used for infection and the CD4^+^ T cell subset studied.

Up until now, investigations of *T. cruzi* infection in mice models have focused on only one CD4^+^ subset, either T_reg_ or Th17, but none have studied both CD4^+^ subsets in the same experimental model. Furthermore, they have all been performed using susceptible model/*T. cruzi* strain combinations, that is, BALB/c infected with the Y strain or C57BL/6 with the Tulahuén strain. Investigations exploring the role that distinct CD4^+^ T cell subsets may play in controlling *T. cruzi* infection are thus needed, particularly in non-susceptible models that control the infection more efficiently.

We performed a comparative study that included analysis of the Th1, T_reg_ and Th17 cell markers in mice models both susceptible and non-susceptible to infection by the *T. cruzi* Y strain. Mice were infected with either low or high parasite loads and were examined throughout the acute and chronic phases of the disease. Our results suggest that a combination of Th1 and T_reg_ responses in the hearts of non-susceptible C57BL/6 mice acutely infected with the Y strain helps to control infection and enhance survival, whereas in susceptible BALB/c mice the combined Th1 and Th17 response protects mice from death only if the parasite inoculum is low. Moreover, we observed a Th17 response in the hearts of BALB/c mice infected with high numbers of the Y parasite strain, associated with high levels of IL-6, which may be responsible for the enhanced mortality observed during the acute phase.

## Results

### Susceptibility of the Mouse Strains to *Trypanosoma cruzi* Infection

**Figure 1:**
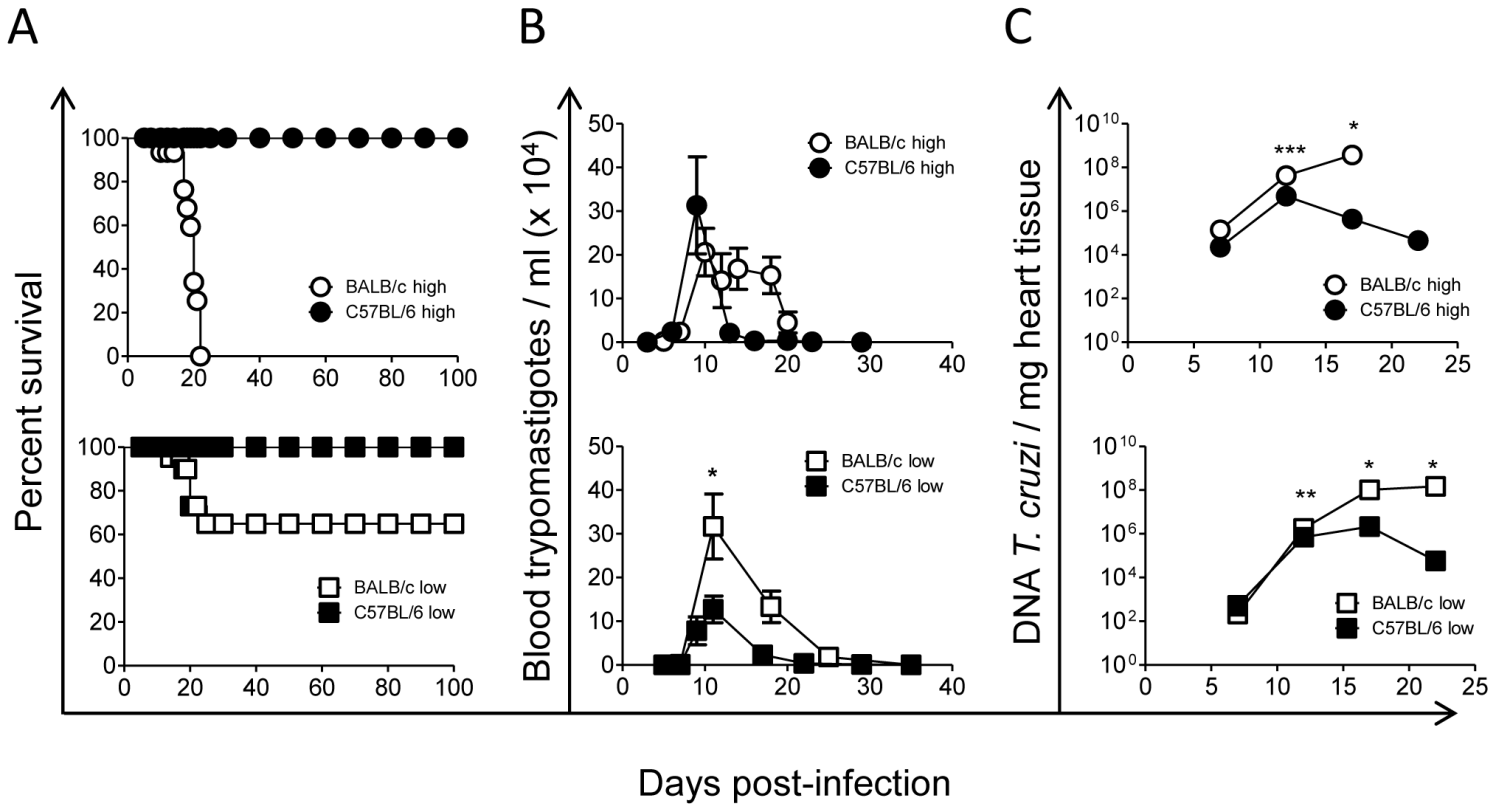
BALB/c mice infected with *T. cruzi* showed higher parasite loads and lower survival rates than infected C57BL/6 mice. BALB/c mice were infected with high inoculum (open circle) or low inoculum (open square). C57BL/6 mice were infected with high inoculum (filled circle) or low inoculum (filled square). (**A**) Survival was monitored from 0 to 100 d.p.i., (**B**) parasitemia was monitored from 0 to 35 d.p.i., and (**C**) the parasite load in heart tissue was determined at 7, 12, 17 and 22 d.p.i. in mice infected with high inoculum (top) or low inoculum (bottom) by extrapolation with parasite DNA standards. Data represent the results of at least two independent experiments performed with 3 mice per experimental group. Statistically significant differences between BALB/c and C57BL/6 mice are shown: *p<0.05, **p<0.01 and ***p<0.001.

All BALB/c mice, but no C57BL/6 mice, succumbed to infection from a high inoculum of the *T. cruzi* Y strain (figure 1A top), despite the fact that similar levels of parasitemia were reached in both mouse strains (figure 1B top). Moreover, BALB/c mice showed a significantly higher parasite load in their hearts than C57BL/6 mice at 12 (10 fold) and 17 (10^3^ fold) d.p.i. (figure 1C top). Even more interestingly, C57BL/6, but not BALB/c mice, showed an efficient clearance of heart parasites by day 17 post-infection (figure 1C top). These differences in parasite load and control in the heart may explain the differences in survival between these mouse strains, which agrees with previous results indicating that C57BL/6 are more resistant than BALB/c to infection with the Y parasite strain [24].

When mice were infected with low inoculum, 60% of the BALB/c and all of the C57BL/6 mice survived (figure 1A bottom). Both parasitemia (figure 1B bottom) and heart parasite load (figure 1C bottom) were significantly higher in BALB/c mice than in C57BL/6 mice at this inoculum level, indicating that the outcome of the infection depends on both the hosts’ genetic background and inoculum size.

### Effect of Infection on Thymic T_reg_ Cells

**Figure 2:**
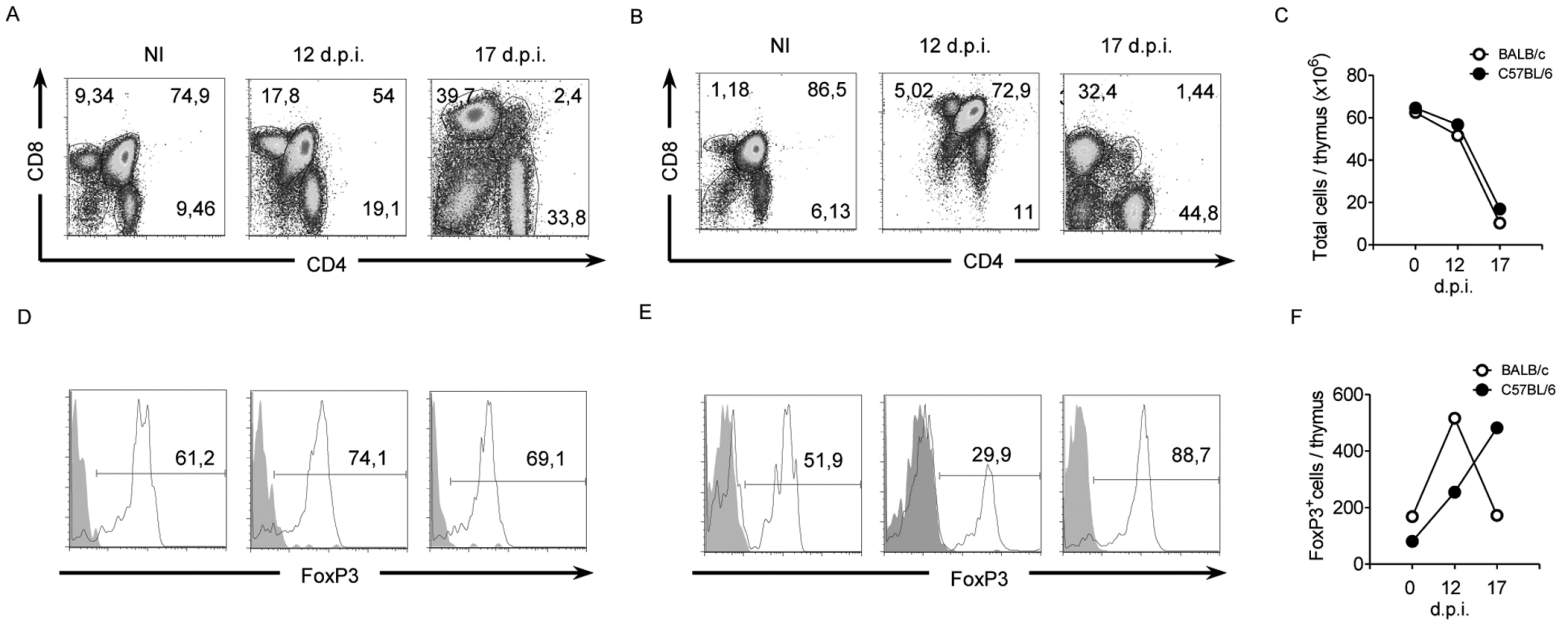
Infected C57BL/6 mice show higher numbers of nT_reg_ cells than infected BALB/c mice. Non-infected (NI) BALB/c and C57BL/6 mice and mice infected with high inoculum were sacrificed at 0 (NI), 12 and 17 d.p.i. Thymocytes were counted and stained with antibodies against cell surface molecules and intracellular markers and analyzed with a flow cytometer. Data from BALB/c and C57BL/6 mice are represented by open circles and filled circles, respectively. (**A**) Anti-CD4 and anti-CD8 antibody staining of thymocytes from non-infected (NI) BALB/c mice at 12 and 17 d.p.i. (**B**) Anti-CD4 and anti-CD8 antibody staining of thymocytes from non-infected (NI) C57BL/6 mice at 12 and 17 d.p.i. (**C**) Total number of thymocytes per thymus in BALB/c (open circles) and C57BL/6 mice (filled circles). (**D**) Staining of the gated CD4^+^CD25^+^ population with anti-FoxP3 antibody in BALB/c mice. (E) Same as “D” for C57BL/6 mice. (F) Total number of FoxP3^+^ cells per thymus. Data represent the results of at least two independent experiments performed with samples pooled from 3 mice per experimental group.

Thymic atrophy has been previously reported as being associated with *T. cruzi* infection and it has been suggested that it plays a role in the pathology of Chagas disease [25]. We analyzed the effect of infection on thymic T_reg_ cells. Thymuses were removed from mice infected with high inoculum at different time points, and analyzed using flow cytometry. Both BALB/c and C57BL/6 mice showed similar depletion patterns of double positive T cells (DP, CD4^+^CD8^+^) and a gradual increase in the percentage of single positive CD4^+^ and CD8^+^ cells (figures 2A and B, respectively). A decrease in the total number of cells per thymus (figure 2C) was also detected, as previously reported [26]. Further analysis of the CD4^+^CD25^+^ gated T cell subset showed that, in contrast with the strong thymocyte depletion, the percentage and number of thymic FoxP3^+^ T_reg_ increased at 12 d.p.i. in both mouse strains (figures 2D and 2E, respectively). Moreover, absolute T_reg_ cell numbers also increased in the thymus at 12 d.p.i. in both strains of mice, and were even higher in C57BL/6 mice at 17 d.p.i. (figure 2F). Further experiments performed at the low inoculum level showed a delay in thymic depletion in both mouse strains but similar patterns regarding T_reg_ cell dynamics (data not shown).

### T lymphocyte Infiltration and Immune Response in Heart Tissue during Acute *Trypanosoma cruzi* Infection

**Figure 3:**
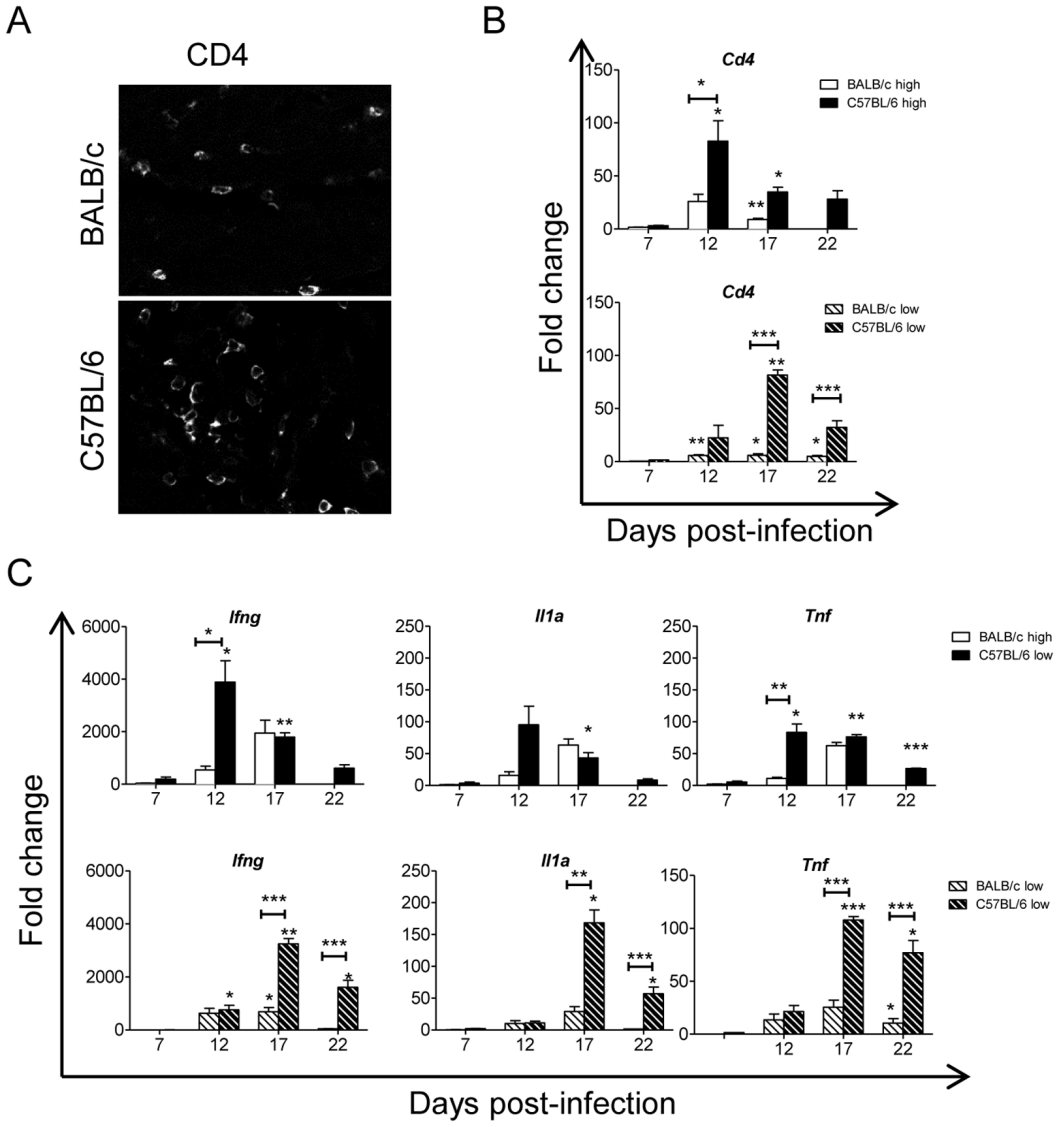
Infected C57BL/6 mice showed greater numbers of cardiac infiltrating CD4^+^ T cells and more inflammation than infected BALB/c mice. BALB/c mice were infected with high inoculum (open bar) or low inoculum (open dashed bar). C57BL/6 mice were infected with high inoculum (filled bar) or low inoculum (filled dashed bar). (**A**) Immunofluorescence staining of heart tissue sections from BALB/c and C57BL/6 mice infected with high inoculum with anti-CD4 antibody at 14 d.p.i. (magnification: 630×). (**B**) Quantitative RT-PCR of total heart tissue RNA from non-infected (NI) mice and mice infected with either high inoculum (top) or low inoculum (bottom) at 7, 12, 17 and 22 d.p.i., utilizing the *Cd4* probe. (**C**) as for “B” utilizing the *Ifng, Il1a* and *Tnf* probes. Data were normalized with respect to NI mice (Fold change: 1) and represent at least two independent experiments performed with 3 mice per experimental group. Statistically significant differences between infected and non-infected mice (0 d.p.i.) and between BALB/c and C57BL/6 mice under each treatment are shown: *p<0.05,**p<0.01 and ***p<0.001.

In humans the heart is one of the organs most severely affected by *T. cruzi* infection [27]. We thus evaluated lymphocyte infiltration in heart tissue sections by immunofluorescence microscopy at 14 d.p.i. in both strains of mice infected with the high inoculum. Figure 3A shows that CD4^+^ T cell infiltration was higher in infected C57BL/6 hearts than in BALB/c hearts. This was confirmed and measured by quantitative RT-PCR of heart mRNA utilizing a *Cd4* probe in mice infected at the high inoculum level (figure 3B).

In addition, in heart tissue of mice infected with high inoculum, mRNA expression of Th1 and inflammatory cytokines, such as IFN-γ, IL-1α and TNF was higher in C57BL/6 than in BALB/c mice (figure 3C top). C57BL/6 mice infected with low inoculum also showed higher levels of IFN-γ, IL-1α and TNF than similarly infected BALB/c mice, although the kinetics were delayed with respect to mice infected with the high inoculum (figure 3C bottom). Interestingly, C57BL/6 mice infected with high inoculum, showed a gradual decrease in Th1 and inflammatory cytokine expression associated with the control of the infection, whereas these parameters increased continually in BALB/c mice inoculated at this same level until 17 d.p.i., just before their death.

### Th17 and T_reg_ Cell Infiltration in Heart Tissue during Acute *Trypanosoma cruzi* Infection

**Figure 4:**
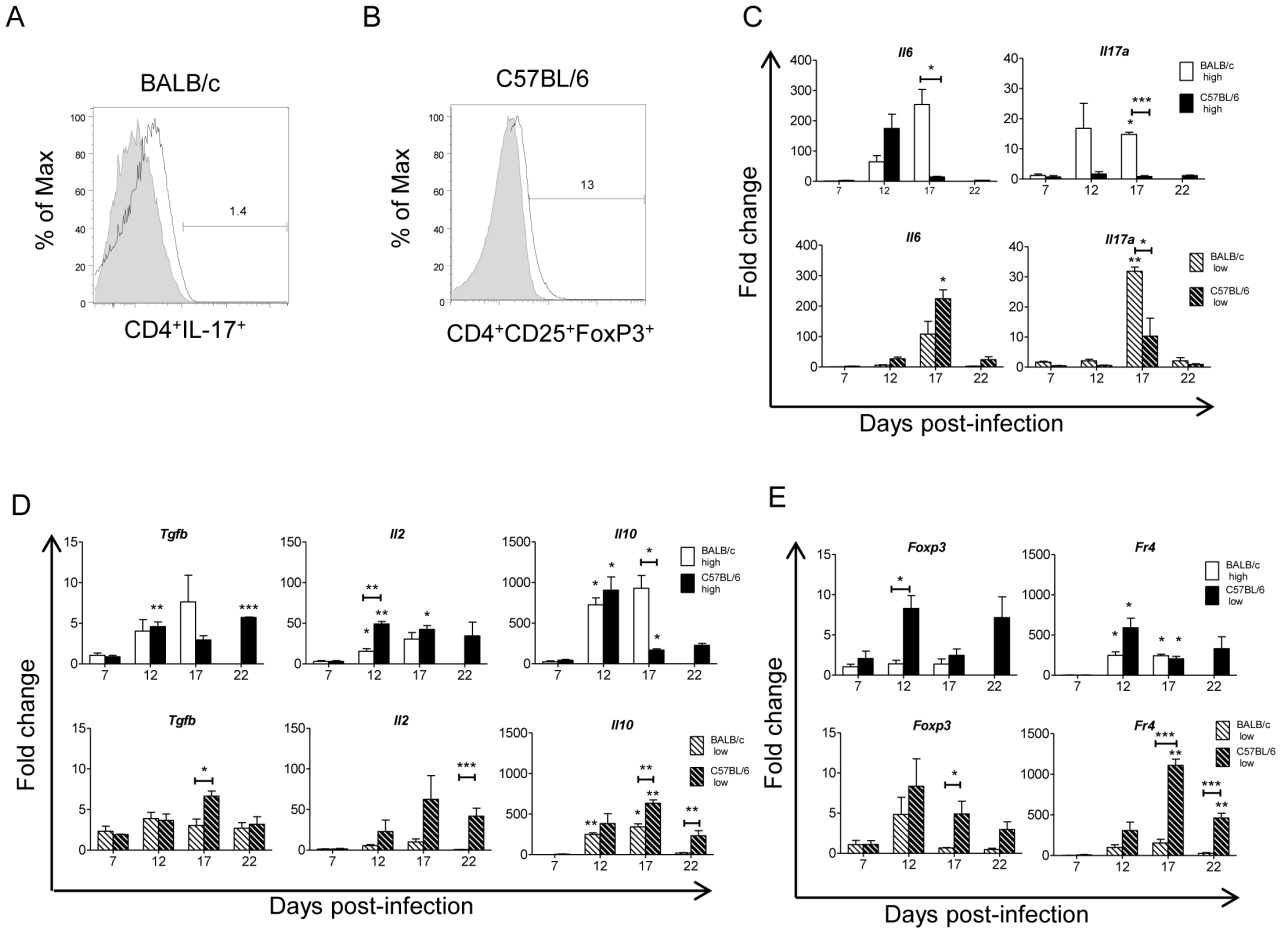
Th17 and T_reg_ cells were isolated from the hearts of BALB/c and C57BL/6 mice, respectively. BALB/c mice were infected with high inoculum (open bar) or low inoculum (open dashed bar). C57BL/6 mice were infected with high inoculum (filled bar) or low inoculum (filled dashed bar). (**A**) Percent of CD4^+^IL-17^+^ cells isolated from the hearts of BALB/c mice infected with high inoculum at 17 d.p.i. (**B**) Percent of CD4^+^CD25^+^FoxP3^+^ cells isolated from the hearts of C57BL/6 mice infected with low inoculum at 17 d.p.i. (**C**) Quantitative RT-PCR from total mouse heart tissue RNA from non-infected (NI) mice and mice infected with either high inoculum (top) or low inoculum (bottom) at 7, 12, 17 and 22 d.p.i., utilizing *Il6 and Il17a* probes. (**D**) as for (C) but utilizing *Tgfb, Il10* and *Il2* probes. (**E**) as for (C) but utilizing *Foxp3* and *Fr4* probes. For “A” and “B” the data represent the results from two experiments performed with 25 and 35 mice per group, respectively. For “C”, “D” and “E” the data were normalized with respect to NI mice (Fold change: 1) and represent the results from at least two independent experiments performed with 3 mice per experimental group. Statistically significant differences between infected and non-infected mice (0 d.p.i.) and between BALB/c and C57BL/6 mice under each treatment are shown: *p<0.05,**p<0.01 and ***p<0.001.

To investigate whether or not T_reg_ and/or Th17 infiltrate cardiac tissue, we isolated CD4^+^ T cells from the hearts of 25–35 mice infected either at the low or the high inoculum level, at different d.p.i. and analyzed the phenotypes of the CD4^+^ populations by flow cytometry. Interestingly, in BALB/c mice infected with the high inoculum, a small but significant proportion of CD4^+^ IL-17^+^ cells were detected at 17 d.p.i. (figure 4A) while no Foxp3 staining was observed (data not shown). In contrast, in C57BL/6 mice infected with the low inoculum, 70.3% of the CD4^+^ isolated cells were CD4^+^CD25^+^, which could correspond either to activated T cells or T_reg_ cells at 17.d.p.i. Furthermore, staining with anti-FoxP3 showed that 13% of the CD4^+^CD25^+^ gated population were Foxp3^+^ (figure 4B). Thus, our results showed that in non-susceptible C57BL/6 mice, T_reg_ cells infiltrate the heart at detectable levels whereas in susceptible BALB/c mice it is the Th17 cells that infiltrate this organ.

We could not recover enough CD4^+^ T_reg_ or Th17 subsets at any other d.p.i. or treatment to allow conclusive evidence. This would have required the sacrifice of many more than 35 mice per treatment, which was non-viable for ethical reasons.

To avoid that limitation and confirm these results, we analyzed markers associated with Th17 cells in the hearts of infected mice by qRT-PCR. IL-6, which is required for Th17 differentiation, was highest in BALB/c mice at 17 d.p.i. when infected with the high inoculum (figure 4C top). At the low inoculum level, however, IL-6 increased at 12 d.p.i. returning to base levels by 17 d.p.i. in both strains of mice (figure 4C bottom). IL-17 was significantly higher at 17 d.p.i. in BALB/c mice compared to C57BL/6 mice infected at either level (figure 4C top and bottom, respectively). Thus, the significant increase in IL-6 and IL-17 in BALB/c mice infected at the higher level could be a cause of the mortality observed. We also analyzed gene expression of cytokines involved in T_reg_ differentiation and function. TGF-β, IL-2 and IL-10 were observed in both strains of mice infected with the high inoculum at 12 d.p.i. (figure 4D top). At 17 d.p.i., however, TGF-β, IL-2 and IL-10 expression decreased in C57BL/6, whereas they further increased in BALB/c mice. This occurred concurrently with the increase of Th1 cytokines in this mouse strain (figure 4D top and figure 3C top). C57BL/6 mice infected at the low inoculum level showed higher IL-10 and TGF-β expression than BALB/c, with maximum differences in gene expression between the two mouse strains occurring at 17 d.p.i. (figure 4D bottom).

In addition, gene expression of T_reg_ cell markers (*Foxp3* and *Fr4*) in heart tissue was highest at 12 d.p.i. in C57BL/6 mice infected with the high inoculum, but interestingly, there was no significant difference in gene expression of these markers between infected and non-infected BALB/c mice (figure 4E top). Similarly, in mice infected at the low level, T_reg_ cell marker expression was higher in C57BL/6 compared to BALB/c mice at all the d.p.i.’s studied (figure 4E, bottom). Thus, T_reg_ cell marker expression in the heart was much higher in the resistant C57BL/6 strain.

### Systemic Immune Response during Acute *Trypanosoma cruzi* Infection

**Figure 5:**
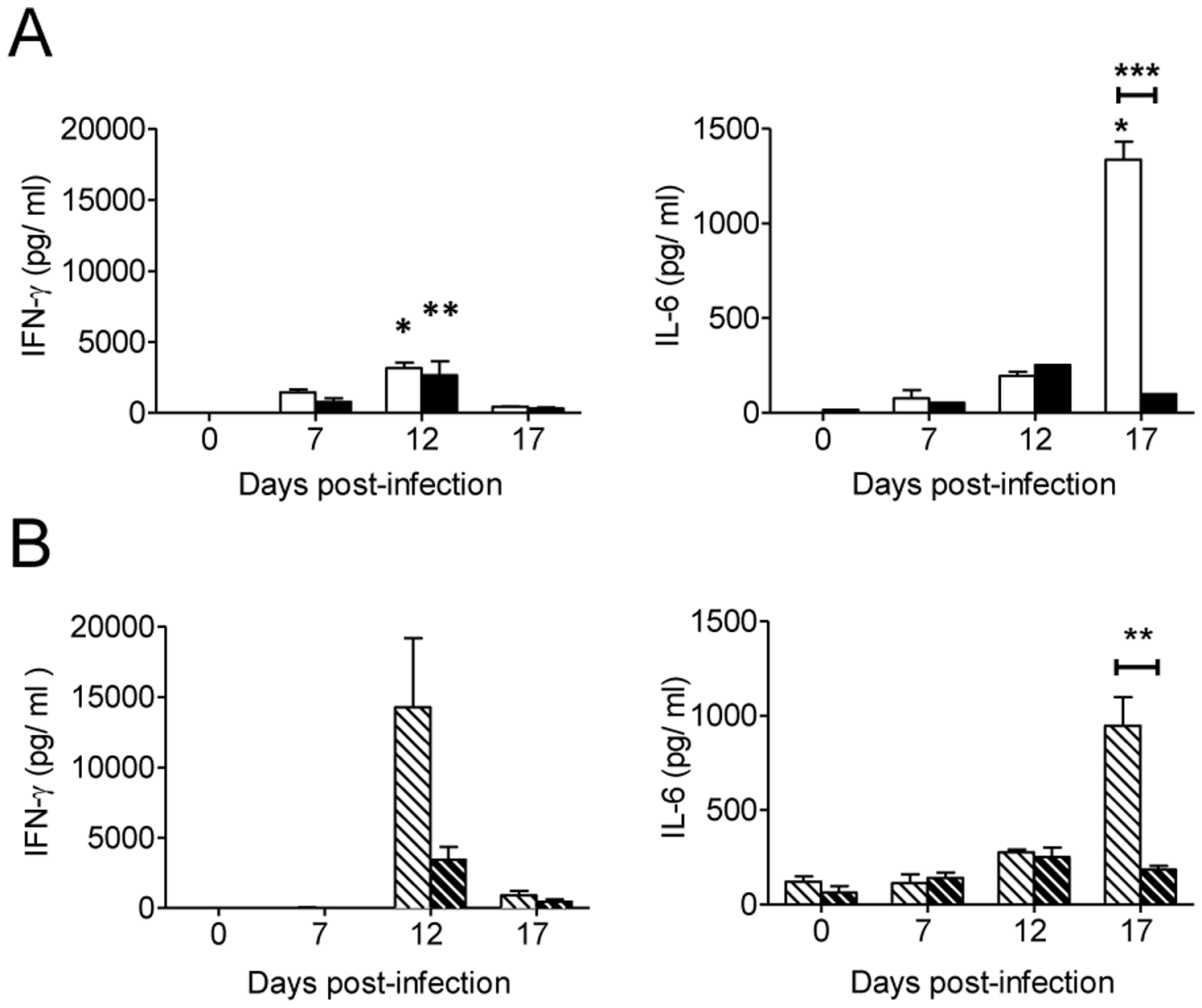
IL-6 serum concentration significantly increased in infected BALB/c mice. BALB/c mice were infected with high inoculum (open bar) or low inoculum (open dashed bar). C57BL/6 mice were infected with high inoculum (filled bar) or low inoculum (filled dashed bar). IFN-γ and IL-6 concentrations were determined from sera extracted from non-infected (NI) mice and mice infected with (**A**) high inoculum or (**B**) low inoculum at 0 (NI), 7, 12 and 17 d.p.i. Data represent the results of at least two independent experiments performed with 3 mice per experimental group. Statistically significant differences between infected and non-infected mice (0 d.p.i.) and between BALB/c and C57BL/6 mice under each treatment are shown: *p<0.05,**p<0.01 and ***p<0.001.

Serum cytokine concentration was determined as a measure of the systemic response against infection. Several cytokines, such as the granulocyte macrophage colony-stimulating factor (GM-CSF), IL-1α, TNF-α, IL-10, IL-17A, IL-2, IL-4 and IL-5, showed a slight but non-significant increase in their serum concentrations upon infection (data not shown). However, in both strains of mice, a significant increase in IFN-γ serum concentration was observed at 12 d.p.i in mice infected with the high inoculum compared to non-infected mice (figure 5A left). Notably, IL-6 serum concentration was significantly higher at 17 d.p.i. in infected BALB/c mice compared to both healthy mice and C57BL/6 mice infected at both inoculum levels (figure 5A and 5B right).

### Parasite Burden and T lymphocyte Infiltration in Heart Tissue during Chronic *Trypanosoma cruzi* Infection

**Figure 6:**
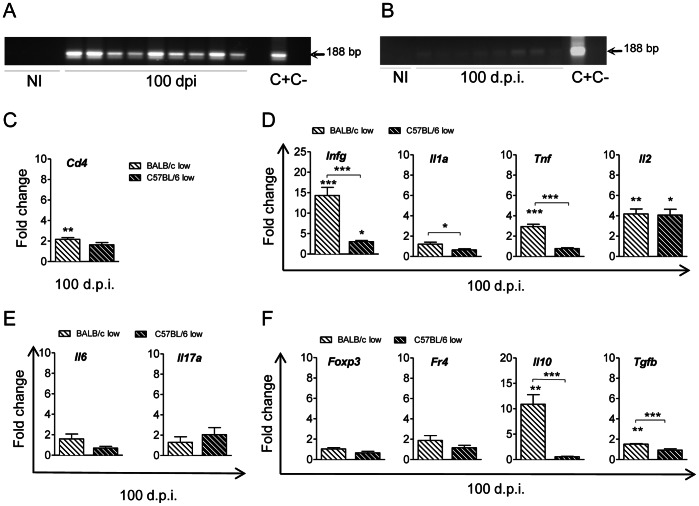
Infected BALB/c mice show a greater parasite load and more inflammation during the chronic phase than infected C57BL/6 mice. BALB/c and C57BL/6 mice were infected with the low inoculum and sacrificed at 100 d.p.i. (**A**) Specific *T. cruzi* PCR with DNA from the hearts of non-infected (NI) and infected BALB/c mice at 100 d.p.i.; parasite DNA was used as a positive control (C+) and H_2_O as a negative control (C-). (**B**) As for “A” but for C57BL/6 mice. Quantitative RT-PCR from total heart tissue RNA of infected BALB/c mice (open dashed bars) and C57BL/6 mice (filled dashed bars) utilizing: (**C**) a *Cd4* probe, (**D**) *Ifng, Il1a, Tnf* and *Il2* probes, (**E**) *Il6* and *Il17a* probes and (**F**) *Foxp3*, *Fr4*, *Il10* and *Tgfb* probes. Data represent the results of at least two independent experiments performed with 3 mice per experimental group and were normalized with respect to NI mice (Fold change: 1). Statistically significant differences between infected and non-infected mice and between BALB/c and C57BL/6 mice under each treatment are shown: *p<0.05,**p<0.01 and ***p<0.001.

To analyze the immune response during the chronic phase, mice infected with the low inoculum were sacrificed at 100 d.p.i. and parasite persistence was analyzed by PCR with *T. cruzi* specific probes. Surviving BALB/c mice (figure 6A) showed stronger PCR-amplified *T. cruzi* DNA signals than C57BL/6 mice (figure 6B), suggesting that they harbored a higher number of parasites during this phase.

The analysis of immune cell markers during the chronic phase showed a low but significant CD4^+^ T cell infiltration in BALB/c mice whereas these T cells were not detected in infected C57BL/6 mice (figure 6C). CD4^+^ infiltration in infected BALB/c mice was associated with a higher expression of Th1 and inflammatory cytokines, such as IFN-γ TNF and IL-2, and to a lesser extent IL-1α, compared to that of non-infected mice (figure 6D). In infected C57BL/6 mice, however, only IFN-γ and IL-2 were detected at higher levels than in non-infected mice, although this difference was much lower than that found between infected and non-infected BALB/c mice (figure 6D).

The low number of CD4 cells present in the mouse hearts during the chronic phase meant that it was impossible to recover enough of them for analysis. Neither could we detect significant changes in Th17 associated genes such as IL-6 and IL-17 expression (figure 6E) or the expression of T_reg_ associated cell markers (FR4 and FoxP3) in chronically infected BALB/c and C57BL/6 mice, compared to healthy mice (figure 6F). Nonetheless, infected BALB/c mice showed a 10 fold increase in IL-10 and about 2 fold in TGF-β expression over non- infected mice of the same age, which was not observed in infected C57BL/6 mice (figure 6F). None of the values of any of the other cell markers that were analyzed in the acute phase showed significant differences between infected and healthy mice of the same age during the chronic phase (data not shown). Our results thus indicate that susceptible mice who survive the acute infection and reach the chronic phase maintain parasites in their hearts that illicit a combined Th1 and regulatory-like response, but not a Th17 response.

## Discussion

The identification of the key factors that determine survival of *T. cruzi* infection in mice as well as the mechanisms controlling infection in the asymptomatic chronic phase of the infection is crucial in order to develop novel strategies to fight Chagas disease. The influence of host genetic background on the susceptibility to T. cruzi infection has been documented both in human and mice [28], [29], [30]. On the other hand, the role of different CD4+ T cell subsets has been reported in experimental T. cruzi infection in mice, but always utilizing susceptible host models of infection. But, to date, a simultaneous comparative analysis of CD4+ T cell subsets in susceptible and resistant hosts has not been performed. Many studies have focused on the Th1/Th2 balance during the acute and chronic phases, but there are only a few reports that discuss the role of other CD4^+^ T cell subsets such as T_reg_ and Th17 cells. Even then, these reports only studied one CD4^+^ T cell subset at a time; either T_reg_ or Th17 cells, and only in susceptible *T.cruzi*/mouse strain combinations, thus providing an incomplete picture of the immunopathogenesis of this complex disease. In this investigation, we undertook a comprehensive study of the dynamics of T cell subsets, analyzing the immune response during both the acute and chronic phases of the experimental infection and furthermore, comparing susceptible and non-susceptible mice infected at two different parasite inoculum levels. This analysis may thus provide some clues as to how these T cell populations may influence the outcome of this disease.

Our results indicate that in the BALB/c-Y strain susceptible model, the higher the parasite inoculum the lower the survival rate, irrespective of the actual parasitemia. Thus, mortality seems to be related specifically to heart parasite load. Heart parasite load was much higher in BALB/c (several logs) than in C57BL/6 mice. Furthermore, C57BL/6, but not BALB/c mice, showed an efficient clearance of heart parasites by day 17 post-infection. Thus, C57BL/6, but not BALB/c mice, are able to control parasite replication in the heart and thus have a better chance of surviving the disease. This agrees with previous results indicating that C57BL/6 are more resistant than BALB/c to infection with the *T. cruzi* Y strain [24].

Moreover, we found important differences in the immunological responses in the hearts of infected mice between susceptible and resistant mouse strains. CD4^+^ T cell infiltration was highest at 12 d.p.i. in C57BL/6 mice infected at the high inoculum level, whilst in mice infected with the low inoculum the time course of cardiac infiltration was delayed to 17 d.p.i. CD4^+^ T cell recruitment into the heart and the expression of Th1 and inflammatory cytokines (such as IFN-γ and TNF) was significantly higher in C57BL/6 mice compared to BALB/c mice at 12 d.p.i. but later decreased at 17 and 22 d.p.i. This coincided with a decrease in parasite load in C57BL/6 mice associated with their survival, thus indicating that a strong infiltration of Th1 in the heart protects against infection during the acute phase of the disease. This agrees with observations concerning the protective role of the Th1 response in controlling *T. cruzi* proliferation during the acute phase, exemplified by the fact that mice deficient in either the IFN-γ [6] or the IFN-γ receptor [7] are highly susceptible to infection.

Inflammation has been considered detrimental for the outcome of Chagas disease [31]. However, C57BL/6 mice, despite showing much greater inflammation than BALB/c mice, controlled the infection and survived. This suggests that although inflammation may control parasite replication, it must be somewhat controlled in order to avoid excessive damage.

We observed thymic atrophy and subsequent CD4^+^CD8^+^ T cell depletion during the acute phase of infection in both strains of mice, as for previous reports [26]. However, since BALB/c, but not C57BL/6 mice, showed high mortality, it is likely that severe thymic atrophy does not determine the outcome of the infection. Despite the depletion of CD4^+^CD8^+^ T cells, we observed an increase in the number of thymic CD4^+^CD25^+^FoxP3^+^ T_reg_ at 12 d.p.i. However, the absolute number of CD4^+^CD25^+^FoxP3^+^ T_reg_ only continued to increase in resistant C57BL/6 mice., This was observed even at 17 d.p.i. when thymocyte depletion was maximal, suggesting that T_reg_ can control excessive inflammatory responses thus counteracting some of the detrimental effects of *T.cruzi* infection.

We would like to note that a previous study has indicated that T_reg_ decreased in the thymus upon T. *cruzi* infection [32]. This apparent inconsistency with our results could be ascribed to the use of a different *T. cruzi* strain as well as the time point analyzed. This suggests that the kinetics of the different responses in experimental *T. cruzi* infection should be taken into account, since these could radically change depending on the time point analyzed. It is also worth mentioning that the origin of the T_reg_ cells found in the thymus during acute infection could be either thymic (nT_reg_) or iT_reg_ cells that have re-entered the thymus from the periphery [33], [34]. Thus, the origin of these T_reg_ cells should be further investigated.

In this study we were able to isolate, for the first time, T_regs_ from the inflammatory heart infiltrate from infected C57BL/6, but not BALB/c, mice. These results indicate that in non-susceptible C57BL/6 mice, T_reg_ responses are generated that likely control the excessive and potentially pathogenic inflammation produced by the strong Th1 response in the heart. Our results suggest that the combined action of Th1 and T_reg_ responses observed in C57BL/6 mice could be protective during the acute phase of infection by combining an effective anti-parasite response with limited damage.

In accordance with our results, several clinical studies have indicated that peripheral T_reg_ cell numbers increased in asymptomatic patients in comparison with Chagas disease patients with overt cardiac pathology [14], [15], [16], suggesting that the regulatory response plays a protective role. However, the results obtained in mouse experimental models are more complex. Thus, when C57BL/6 mice highly susceptible to the Tulahuén or the Brazil strain were infected and then treated with anti-CD25 antibodies to deplete T_regs_, the results suggested that these cells played a limited role in the control of *T. cruzi* infection in muscle [17] and heart [18]. In contrast, BALB/c mice, infected with a sub-lethal inoculum of the Y strain and then depleted of T_regs_ by treating them with anti GITR (glucocorticoid-induced TNFR-related protein) rather than anti-CD25 antibodies, showed an increase in heart parasite burden and host susceptibility [19]. However, the results of the above mentioned experiments should be interpreted with caution, since anti-CD25 antibodies are not specific for T_reg_ cells and may eliminate other types of activated T cells.

On the other hand, IL-6 may play an important role in determining the outcome of the disease. Both serum IL-6 concentration and IL-6 expression in the heart significantly increased at 17 d.p.i. in BALB/c, but not C57BL/6 mice, infected at the high inoculum level. This means that the strong IL-6 expression in heart tissue and high concentrations of systemic IL-6 secretion could be linked to the high mortality observed in BALB/c mice during the acute phase. High IL-6 levels in infected BALB/c mice may lead to Th17 cell differentiation while inhibiting T_reg_ cell development [13]. Accordingly, infiltrating Th17 cells were only isolated from infected BALB/c hearts and only at 17 d.p.i. Five days later all the BALB/c mice infected at the high level died, showing that high Th17 responses may be associated with uncontrolled parasite replication in the heart leading to death in this mouse strain. Other authors have demonstrated, using anti-IL-17 antibody treatment, that IL-17 plays a protective role in BALB/c mice infected with a low inoculum of the Y strain, although this was attributed to the Th17-mediated suppression of excessively pathogenic Th1 responses in the heart [21].

Investigations undertaken using a different *T. cruzi* strain, Tulahuén, produced higher mortality in C57BL/6 than in BALB/c mice. Thus, in this experimental model C57BL/6 mice are more susceptible to infection than BALB/c mice [35], [36]. In this context, susceptible C57BL/6 mice became even more susceptible to infection when IL-17 [22] and the IL-17 receptor [23] were genetically eliminated. In addition, IL-6-deficient C57BL/6 mice were more susceptible to infection with the Tulahuén strain than wild type mice due to deficient lymphocyte recruitment [37]. In this experimental model, C57BL/6-Tulahuén, mortality is likely due to fatal liver damage caused by the differential modulation of hepatic Toll-like receptors, rather than cardiac injury [35]. Thus, it seems that different *T. cruzi* strains may exhibit diverse pathogenic mechanisms which attack different host organs, thus affecting the outcome of the disease [38].

Taken together, all of the above clearly indicates that the IL-6/Th17 or Th1/T_reg_ responses may be either protective or pathogenic depending on the *T.cruzi*-mouse strain combination. Overall, however, kinetic studies suggest that cardiac T cell mobilization is quicker in C57BL/6 than in BALB/c mice. This influences the extent of parasite replication and the infiltration of T_reg_ or Th17 cells in the heart.

In previous studies we observed greater numbers of MDSCs infiltrating the heart in susceptible BALB/c compared to resistant C57BL/6 mice infected with a high Y strain inoculum [39], [40]. Here we found that Th17 cells were also infiltrating BALB/c cardiac tissue, and that the high mortality produced in these mice when infected with a large parasite load was associated with high levels of IL-6. Thus, there is likely some correlation between IL-6, Th17 cells and MDSCs in susceptible mice, associated with high mortality. In addition, we found that heart infiltrating heterogeneous CD11b^+^ cells isolated from BALB/c mice at 21 d.p.i. expressed IL-6 and IL-10 [39]. Since the number of these cytokines increased in BALB/c mice with scarce infiltrating CD4^+^ T cells it is tempting to speculate that in the susceptible model lL-6 and IL-10 are being produced by a subset of infiltrating CD11b^+^ cells.

In another type of cardiac disease; experimental autoimmune myocarditis (EAM), the IL-6/IL-17 response seems to be pathogenic. In EAM, IL-6 is critical in the progression from inflammatory myocarditis to fibrotic dilated cardiomyopathy [41]. In addition, IL-17-deficient animals were protected from fatal heart failure and did not develop EAM induced severe dilated cardiomyopathy [42]. Thus, with regard to the role of IL-17 in myocarditis, there do appear to be some similarities between EAM and our susceptible model of *T. cruzi* infection of BALB/c mice with the cardiotropic Y strain.

It is interesting that during the chronic phase, heart IFN-γ and IL-10 expression were higher in BALB/c than in C57BL/6 mice. This may indicate that in chronically infected BALB/c mice, in addition to a residual Th1 response against persisting parasites, regulatory cytokines are expressed in heart tissue, albeit in the absence of Foxp3 expressing T_reg_ cells. Nevertheless, BALB/c mice, despite having a detectable parasite burden and inflammatory cytokines at 100 d.p.i., did not show any external symptoms of the disease. This may be due to the balancing effect of anti-inflammatory IL-10, although more experiments are needed to investigate this hypothesis.

In summary, our work describes for the first time an association between the presence of Treg cells isolated from the heart of Trypanosoma cruzi infected mice with resistance to infection with the Y parasite strain. We also describe that the presence of Th17 cells is associated with resistance to infection in susceptible BALB/c mice. However, the regulatory response seems to be more beneficial than the Th17 response for controlling infection with high parasite inocula. Although there are reports on Treg and Th17 in the literature, we believe our contribution is important since those studies were performed only on susceptible models, and concluded that Treg cells play a limited role in the control of infection, while Th17 cells protect mice from infection. Moreover, our results put a word of caution when analyzing the nature and importance of the various CD4+ T cells subsets in the mouse models of Chagas disease, since they may be protective or pathogenic depending on the T. cruzi-mouse strains combinations and may help to better understand, the immunopathological responses of such a complex disease. Future experiments will focus on the identification of the cellular sources of the relevant cytokines involved in cardiac inflammation with the aim of designing immune intervention protocols that ameliorate the outcome of the disease.

## Methods

### Ethics Statement

This study was carried out in strict accordance with the European Council Directive [43]. Mice were maintained under pathogen-free conditions at the UAM animal facility. The protocol for the treatment of the animals was approved by the “Comité de Ética de Investigación de la UAM”, Spain. Animals had unlimited access to food and water. They were euthanized in a CO_2_ chamber and all efforts were made to minimize their suffering.

### Parasites and Mice

Young adult (6 to 8-week-old) BALB/c and C57BL/6 female mice were transported from Charles River Laboratories and hosted in a controlled environment. *T. cruzi* Y strain blood trypomastigotes were routinely maintained by infecting IFN-γ receptor deficient mice and purifying them from their blood. Infections at either a high (2×10^3^ trypomastigotes per mouse) or a low (50 trypomastigotes per mouse) inoculum level were performed by intra-peritoneal injection after two weeks of quarantine. Parasitemia was monitored by the Brener method as described in [44].

### Serum Cytokine Measurement

Serum cytokine concentration was determined using beads coupled to fluorescent antibodies specific to different cytokines using the Mouse Th1/Th2 10-plex Flowcytomix Multiplex kit (eBioscience). Samples were analyzed following the directions of the manufacturer in a FACSCanto II Cytometer (Becton Dickinson).

### Removal of Organs and CD4^+^ Magnetic Cell Sorting

Groups of 25 C57BL/6 mice and 35 BALB/c mice were infected with the *T. cruzi* Y strain. The parasitemia was monitored and mice were euthanized at different days post-infection (d.p.i.). Hearts were processed as described in [39]. Briefly, hearts were reperfused with 10 ml of PBS and 1 U/ml heparin after purification of T cell tissue. Groups of 4 hearts were digested with 600 U/ml collagenase II (Worthington, CLS-2) and 60 U/ml of DNAse I in the gentleMACS™ Dissociator following the directions of the manufacturer (Miltenyi Biotec). CD4^+^ cells were isolated with CD4 Microbeads following the directions of the manufacturer (Miltenyi Biotec) giving CD4^+^ isolated cells with 95% cell purity. Thymic cells were obtained by the mechanical disruption of the thymus and passing the resulting material through a 40 µm cell strainer (BD Falcon).

### PCR, Quantitative Real-time PCR and Quantitative Reverse-transcription (RT)-PCR

Heart DNA was isolated using the High Pure PCR Template Preparation Kit (Roche). Heart tissue samples used in PCR reactions contained 100 ng of genomic DNA, and *T. cruzi* was detected using nested PCR [45]. For quantitative PCR, samples were run in duplicate with *T. cruzi* probes [46] and the genomic mouse TNF Taqman probe (Applied Biosystems). The quantity of *T. cruzi* DNA in mouse heart tissue was calculated from the comparative threshold cycle (C_T_) values obtained from *T. cruzi* probes and normalized with respect to the mouse TNF probes. The regression equation resulting from plotting the C_T_ values obtained from serial dilutions starting from 100 pg to 0.001 pg of parasite DNA standard was then used to extrapolate the quantity of parasite DNA in the samples. Results were expressed as pg of *T. cruzi* DNA per mg of heart tissue DNA. Total RNA was extracted from hearts with TRIzol reagent (Invitrogen) following the manufacturers’ instructions. For quantitative RT-PCR analysis, reverse transcription of total RNA was performed using the High Capacity cDNA Archive Kit (Applied Biosystems) and the amplification of different genes encoding clusters of differentiation; (CD)4 (*Cd4*), interferon (IFN)-γ (*Ifng)*, interleukin (IL)-1α (*Il1a*), tumor necrosis factor (TNF, *Tnf*), IL-2 (*Il2*), IL-6 (*Il6*), forkhead box P3 (FoxP3, *Foxp3*), folate receptor (FR)4 (*Fr4*), IL-10 (*ll10*), transforming growth factor (TGF)β (*Tgfb*) and IL-17 (*Il17a*), was performed in triplicate utilizing Taqman probes (Applied Biosystems). The relative quantity of each of the genes was then calculated by the comparative threshold cycle (C*_T_*) method following the manufacturer’s instructions. All quantifications were normalized to the *18S* gene to account for variability in the initial concentration of RNA and in the conversion efficiency of the reverse transcription reaction (ΔC_T_). Finally, all data from samples taken from infected mice were normalized with respect to the values obtained from non-infected mice (ΔΔC_T_). The relative quantity (RQ) was calculated as: RQ  =  2^−ΔΔC^ *_T._*

### Immunofluorescence

Hearts were fixed in 4% paraformaldehyde in PBS solution for 2 h at room temperature, incubated in a 30% sucrose solution overnight at 4°C, embedded in Tissue-Tek O.C.T. compound (Sakura), and frozen. Sections 10 µm thick were then cut and fixed in acetone. The sections were incubated with goat anti–mouse CD4 antibody (BD Pharmingen) at 4°C overnight, and then with anti-goat IgG Alexa Fluor 488 at room temperature for 1 h (BD Pharmingen). Slides were preserved in Prolong Gold Antifade (Invitrogen) and images were obtained using an LSM510 Meta confocal laser coupled to an Axiovert 200 (Zeiss) microscope.

### Flow Cytometry

Flow cytometry was performed as previously described [39]. For IL-17 intracellular staining, cells were previously stimulated with PMA/ionomycin (Sigma) in the presence of Brefeldin A (BD Pharmingen) for 4 h. For FoxP3 and IL-17 intracellular staining, cells were permeabilized with the Cytofix/Cytoperm Kit (BD Pharmingen). FcγRs were blocked with anti CD16/CD32 antibody (Fc block) prior to staining with antibodies coupled to fluorophores. The flow cytometry staining antibodies used were: FITC-conjugated-anti-CD4 (clone RM4-5), PE-conjugated-anti-CD8a (clones 53.6.7), PE-conjugated Rat IgG2a,k, FITC-conjugated Rat IgG2b and Cytofix/Cytoperm Kit from BD Pharmingen; APC-conjugated-anti-CD25 (clonePC61.5), PE-conjugated-anti-FoxP3 (Clone FJK-16s), APC-conjugated-anti-FR4 (clone eBio12A5), APC-conjugated-anti-IL-17 (clone eBio17B7), PE-conjugated Armenian hamster IgG1, AlexaFluor647-conjugated Rat IgG2b and APC-Conjugated-Rat IgG2b, from eBioscience. Samples were analyzed in a FACSCanto II Cytometer (Becton Dickinson) using the FlowJo software (Tree Star, Inc. Oregon Corporation).

### Statistical Analysis

All experiments performed for gene expression analysis were performed in groups of three mice (n = 3) and data are reported as means ± standard error of the mean. A representative experiment of gene expression out of at least two experiments is shown. Statistical significance was evaluated using the Student’s *t*-test (95% confidence interval) with the GraphPad Prism version 5.0 for Windows (GraphPad Software, San Diego California USA, www.graphpad.com). The Welch correction was applied when variances were significantly different.
